# Active Packaging Based on Coupled Nylon/PE Pouches Filled with Active Nano-Hybrid: Effect on the Shelf Life of Fresh Milk

**DOI:** 10.3390/nano11081881

**Published:** 2021-07-22

**Authors:** Valeria Bugatti, Federica Zuppardi, Gianluca Viscusi, Giuliana Gorrasi

**Affiliations:** 1 Department of Industrial Engineering, University of Salerno, Via Giovanni Paolo II 132, 84084 Fisciano, Italy; vbugatti@unisa.it (V.B.); gviscusi@unisa.it (G.V.); 2 Nice Filler s.r.l., Via Loggia dei Pisani 25, 80133 Napoli, Italy; federica.zuppardi@nicefiller.it

**Keywords:** active packaging, layered double hydroxides, milk, shelf life

## Abstract

The study reports on the preparation and characterization of an active packaging based on pouches composed of a coupled system nylon/polyethylene (PE). The PE layer was filled with and active nano-hybrid of layered double hydroxide (LDH) on which it was anchored salicylate, as antimicrobial molecule. The release of the salicylate anchored to the LDH was compared to the release of the molecule free dispersed into the PE and resulted much slower. It was evaluated the efficiency of the active packaging to inhibit *Pseudomonas aeruginosa*, *Escherichia coli*, *Listeria monocytogenes*, *Salmonella typhimurium*, and *Campylobacter*. Global migration tests on the PE active layer, using ethanol (50% *v*/*v*) as food simulant, demonstrated the possibility of such active nanocomposite to be used for food contact being the migration limits in compliance with those imposed from the EU regulation. Fresh milk was packed into the active pouches and pouches with unfilled PE layer, as control. The pH reduction as function of the time, due to the production of lactic acid, resulted much slower in the active packaging. Total bacterial count (TBC) was evaluated on the milk, either packed into the active packaging or the control, up to 50 days of storage at 4 °C. Shelf life of the milk was evaluated using the Gompertz model. It was demonstrated an increasing of the shelf life of milk packaged in active pouches from 6 days up to 10 days.

## 1. Introduction

Milk can be considered a complex mixture of many substances such as water, lipids, proteins, carbohydrates, enzymes, vitamins, and minerals. Because of its composition and the quasi neutral pH, milk is a highly perishable food product characterized by high spoilage potential leading to a rapid deterioration of quality. It can be related to many effects such as the effect of light and oxygen. In fact, they induce oxidation and autoxidation of milk fat, psychrotrophic bacterial activity/enzymatic activity. It can follow a noticeable flavor changes or interaction with the packaging material resulting in product flavor deterioration. Besides, milk safety may be compromised by incomplete destruction of pathogenic microorganisms. Packaging can give a protection from such hazards [1,2]. It covers a number of different functions such as containment, protection, convenience, and communication, the most important being protection [3]. Packaging must protect milk against environmental, physical, chemical (i.e., light, O_2_, moisture), as well as mechanical hazards [4]. A packaging intended for milk must possess the following main characteristics: (i) protection against contaminants, (ii) protection against light and external odors, (iii) physical protection to withstand distribution hazards. Several packaging can be considered for milk storage, for example glass bottles. Glass materials are characterized by fragility and weight, although returnable glass bottles are being considering as an environmentally friendly choice. Paperboard cartons (i.e., Tetrapak, Tetrabrik, Purebrik) are also used for milk packaging. The industrial equipment used to produce such manufactures are expensive and the end use is difficult to manage, post-consumer is difficult to handle as recycling a material made up of various layers of different materials is a rather complex operation. Plastic containers are gaining more and more on the global market for milk storage, the main plastics used in pasteurized milk packaging are HDPE (high density polyethylene), PET (polyethylene terephthalate), PC (polycarbonates), and LDPE (low density polyethylene). Plastic bottles with volumes between 1 and 4 L are widely used to package pasteurized milk. Since unpigmented PE bottles transmit 58–79% of the incident light in the wavelength range 350–800 nm, TiO_2_ at 1–2% is often added producing an opaque bottle. Plastic jugs are extrusion-blow-molded to provide a thin-walled, lightweight, and tough container. An advantage of this type of packaging mainly regards the handle on the bottle, which makes it more convenient to hold respect to paperboard cartons. An innovative plastic packaging could be represented by pouches. These are single-trip packaging, with very light weight, and whose production and distribution costs are incomparable with glass bottles and paperboards packaging. Losses of milk during pouch filling are less than bottle filling and less space is required for packing section and cold storage. The present paper reports the preparation of plastic pouches for milk storage made of coupled layers of LDPE and Nylon 6.6. The internal layer is LDPE filled with an active compound made of layered double hydroxides (LDH) chemically modified with an antimicrobial molecule listed in EC-Directive 10/2011, that is salicylate, while the external layer is a Nylon 6.6 pigmented with TiO_2_ at 1%, in order to preserve the packaged milk from the effect of light, but avoiding the direct contact of the pigmented layer with the liquid. The LDH, selected as nano-carrier, was already widely used either in drug delivery or in food packaging applications highlighting the great potentialities of such anionic clays for increase the shelf life of foods packed in materials hosting such nano-carriers [5,6,7,8,9,10]. The structural characterization of the active layer (PE-Active Filler) was evaluated in terms of degree of dispersion of the layered filler into the polymer and migration in a food simulant, in order to determine the capability of such film for food contact. The bilayer pouches were mechanically characterized. Moreover, the capability of the material to inhibit *Pseudomonas*, *Escherichia coli*, *Listeria*, *Salmonella*, and *Campylobacter* was even evaluated. The pH and acidity evolution (4 °C) up to 35 days and the total bacterial count (TBC) up to 50 days of storage were evaluated on the packaged milk. The shelf life was also evaluated using a mathematical prediction based on the Gompertz equation. The novelty of the present work is represented by the fact that such system can be produced using simple and economic procedures easily scalable on industrial scale. 

## 2. Materials and Methods

Polyethylene (PE), an LDPE with density of 0.92 g/cm^3^ (PRISMA AD PE RIPE RET 91735) was supplied by Frilvam spa, Milano (Italy).

The active filler, with trade name of A1B11^®^ and based on a LDH intercalated with antimicrobial salicylate anion, with a content of 45 wt% of the active molecule, was produced by Nicefiller Ltd. (Naples, Italy), a startup of the University of Salerno. The synthesis was conducted accordingly to a previously reported procedure [11]. The filler was micronized by FPS srl (Piacenza, Italy) to obtain 1–2 micron sized particles. A masterbatch (MB) of PE was obtained by mixing 25 wt% of A1B11^®^ filler, previously dried at 105 °C for 24 h, with 75 wt% of PE in a twin screw extruder at 110 °C and extruding the mixture through a strand die. The strand was cooled, cut, and dried at 70 °C for 3 h. Starting from 35 wt% of MB based on polyethylene (PE) and 25 wt% filler A1B11^®^, rolls (10–12 cm wide) of multilayer PE film (three layers) were prepared using a three-extruder bubble coextrusion plant, with the 8 wt% of final Active Filler in the 10 micron thick PE layer (50 micron total thickness). A control sample, composed of PE and free salicylate at 8 wt% that is the molecule percentage bonded to LDH, simply mixed was prepared in the same conditions used for composites. The PE film was coupled to a 15 micron thick Nylon 6.6 (OPA 15.1100, density 1.15 g/cm^3^), supplied by Maca srl (Benevento, Italy). In the following, the PE filled with the LDH-salicylate will be coded: PE-Active Filler, the total pouches composed of the coupled polymers will be coded: Nylon/PE-Active Filler.

X-ray diffraction (XRD) patterns were taken in reflection with an automatic Bruker diffractometer D8 (Karlsruhe, Germany), using nickel-filtered Cu Kα radiation (Kα = 1.54050 Å) and operating at 40 kV and 40 mA, with a step scan of 0.05° of 2ϑ and 3 sec of counting time. 

The release kinetics of salicylate were followed using a Shimadzu UV-2401 PC spectrometer (Shimadzu, Kyoto, Japan). The tests were performed using 4 cm^2^ rectangular specimens placed into 25 mL of ethanol with 0.9 wt% of tetrabutylammonium chloride and stirred at 100 rpm in an orbital shaker (VDRL MOD. 711 + Asal S.r.l., Milan, Italy). The release medium was withdrawn at fixed time intervals and replenished with fresh medium. The considered band was 230 nm. The experimental results were fitted using the Peppas–Sahlin model (Equation (1)). Such a model takes into account the contribution of two mechanisms: diffusion and relaxation [12] in terms of the coupled effect of the Fickian diffusion and polymer relaxation mechanisms related to an anomalous release process with two phenomena controlling the drug release: [13]

mt/m∞ = K_1_ × t^m^ + K_2_ × t^2m^ (1)

K_1_ is the kinetic constant for Fickian contribution of drug release, K_2_ is the kinetic constant for Case-II contribution, and m a diffusional exponent. The term K_1_ × t^m^ represents Fickian contribution, while the term K_2_ × t^2m^ is related to the Case-II relaxational contribution.

Mechanical properties were evaluated using a dynamometric apparatus INSTRON 4301 (Buckinghamshire, UK). The experiments were carried out at room temperature with the deformation rate equal to 10 mm/min. Data were averaged on quintuplicate.

Overall migration tests were performed in accordance to EN 1186 Migration Testing for Food Contact Materials. Specimens with 1 dm^2^ of surface area (10 cm × 10 cm, 0.10 mm thickness) were put in contact with 100 mL simulant preconditioned at 40 °C, named D1 (Ethanol at 50%), in a borosilicate glass tube closed with a screw cap internally layered with Teflon^®^. The obtained surface/volume ratio was 10 dm^2^/L. The overall migration results were calculated by using 6 dm^2^/kg food (6 dm^2^/L simulant) as conventional EU surface/volume ratio. A known aliquot of the simulant from the contact solution was transferred into a weighted quartz capsule and evaporated to dryness until constant weight. From the differences between the weights, the overall migration was derived in accordance to EN 1186 Migration Testing for Food Contact Materials. The data were averaged on five samples.

The in vitro effect of inhibition against *Pseudomonas aeruginosa ATCC 15442*, *Escherichia coli ATCC 8739*, *Listeria monocytogenes ATCC 13932*, *Salmonella typhimurium ATCC 14028*, *Campylobacter ATCC 33559ATCC 33559ATCC 33559*, provided by 3aLaboratori (Padua, Italy) on the treated PE/Nylon pouches was analyzed following the directive ISO 22196: 2011: such method evaluates the antibacterial activity of treated plastics, surfaces, and other non-porous material [14]. 

pH determinations on packaged milk were carried using a pH-Burette 24 pH-meter equipped with a type 5014T electrode (Crison Instruments, Barcelona, Spain). Data are the average of three replicates.

Acidity °SH/mL determination was carried out on milk by an acidimeter with the Soxhlet-Henkel method (SH/50 cc), using a graduated burette mL 35. Data were evaluated on eight pouches with 100 g of milk.

Total bacteria count (TBC) evaluation in stored milk, as function of time, was conducted using ten grams of milk from each sample aseptically collected and added to 90 mL of saline peptone water. The mixture was homogenized for 10 min in a stomacher 400 (Lab Blender, Seward Medical, London, UK) and 1 mL of homogenate subjected to serial dilutions in the same diluent. Aliquots of 0.1 mL of different dilutions were spread onto the culture media selective for the type of organism (from Oxoid). Microorganisms were quantized with the method based on count of Colonies Forming Units (CFU), by using 25–250 CFUplates^−1^ as range of countable colonies to limit the error due to variability (1, 2, 3). Total bacteria count (TBC) were evaluated by unselective plate count (PCA) agar incubating at 30 °C for 48 h (h). Data were averaged on 8 pouches with 100 g of milk.

The Gompertz equation was used to calculate the shelf life (S.L.) of packaged milk by applying the Equation (2) [15]:

log(CFU) = K + A × exp{−exp{[(μ_max_ × 2.7182) × (λ − t)/A] + 1}} (2)

where K (log(CFU/g)) corresponds to the initial level of bacterial count, μ_max_ represents the maximum growth rate, λ represents the lag phase expressed in days, A concerns the maximum bacteria growth evaluated at the stationary phase, and t is the time (days). Shelf life was determined through the Equation (3):

S.L. = λ − (A × {ln[−ln((log(1 × 10^5^) − K)/A)] − 1})/(μ_max_ × 2.7182) (3)

where 1 × 10^5^ is the acceptability limit for total microbial count of pasteurized milk.

Statistical Analysis was conducted using Origin Lab Software. Results were expressed with standard deviation (SD). ANOVA and Tukey’s tests were carried out using one-way ANOVA and Tukey options in Origin Lab Pro Software (significativity α < 5%). 

## 3. Results

**Figure 1:**
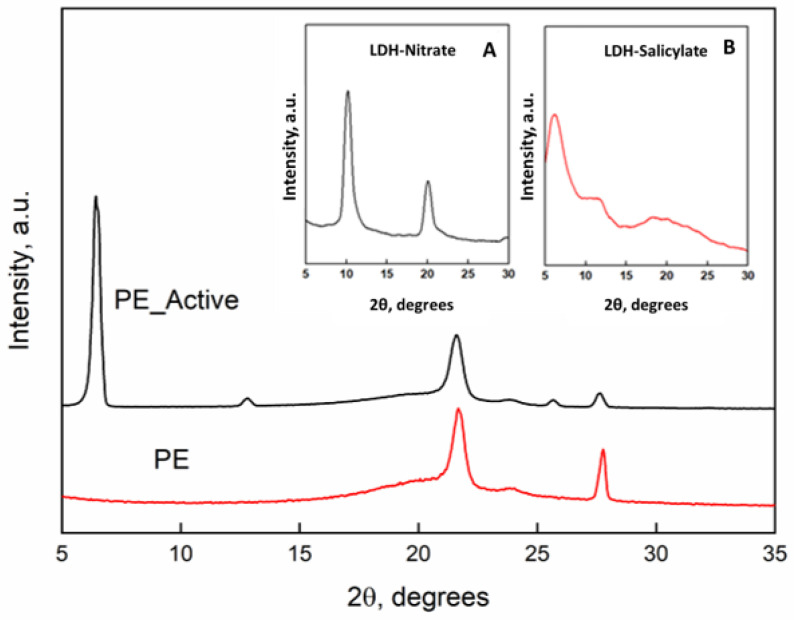
X-ray diffraction patterns of unfilled PE and PE-Active filler. Inset: (**A**) pristine LDH in nitrate form and (**B**) LDH hosting salicylate.

Figure 1 reports the x-rays diffraction patterns of the PE and PE-Active Filler. Inset they were reported the x-rays spectra of the pristine LDH with nitrate anion and the “active filler” that is the LDH in which nitrate (a) was substituted with the salicylate anion (b). The intercalation between the LDH lamellae is clearly demonstrated by the increasing of the basal spacing (d), that corresponds to the interlayers distance, that is 0.86 nm (2θ = 10.2°) for the LDH-nitrate, and becomes 1.63 nm (2θ = 6.02°) for the substitution of salicylate anion with a steric hindrance higher than the nitrate [9]. The diffractometric patterns of the films of unfilled PE and PE filled with the active filler display the typical orthorhombic crystalline cell of PE with the main peaks at 2θ = 21.6° and 2θ = 23.8°. Such structural organization is retained in presence of the active filler. The presence of the peak of the basal spacing of the LDH with the intercalate salicylate at 2θ = 6.02° is also present in the spectrum of the nanocomposite.

**Figure 2:**
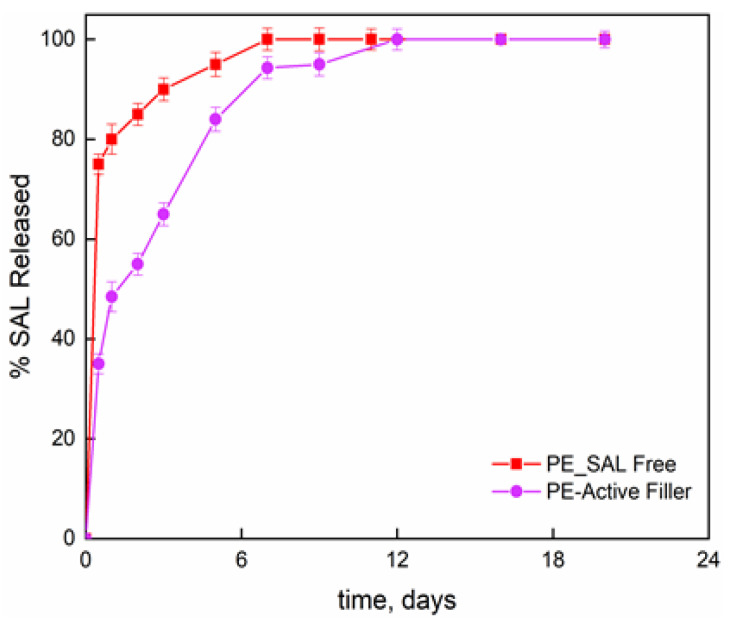
Release of salicylate anchored to LDH in PE-Active Filler and salicylate at 8 wt% free dispersed into PE film.

Figure 2 reports the release fraction of salicylate, as function of the time, for the molecule bonded to the LDH layers and dispersed into the PE film and the molecule simply blended to the PE at the same percentage than the one chemically bonded (8 wt%). The initial release concerns the burst release; then slower release kinetics can be observed until attaining a plateau regime. For salicylate simply blended to the PE, the second release step is attributed to the counter-diffusion of the salicylate ions from the bulk of the material. The total released amount is reached in one week. The film PE-Active Filler displays a release of salicylate in three zones; (i) initial burst step which appears much lower than the one observed for the simply blended filler in PE; (ii) de-intercalation of active molecules; and (iii) counter-diffusion. The released salicylate fraction, at any contact time, is always lower for the active film compared to PE film and the complete release is achieved in 12 days. It is worth underlining that such investigation was performed mainly to have a further proof that salicylate was effectively intercalated between the inorganic layers. 

The evaluated Peppas–Sahlin’s model parameters are reported in Table 1.

A higher value of K_1_ respect to the K_2_ indicates that Fickian diffusion represents the predominant mechanism of molecule release from the polymer film. The negative value of K_2_ for both samples indicated the quasi-absent relaxational contribution and, so, a perfectly Fickian behavior. [16]

Global migration tests were carried out on the film of PE-Active Filler in order to test the suitability of this active layer for food contact. Table 2 reports the global migration in D1 simulant (ethanol at 50% (*v*/*v*), accordingly to EU Regulation) [17,18]. The experimental results prove the suitability of the prepared active material for food contact being the global migration below the migration limits.

In order to evaluate the bacteriostatic activity of the active pouches, the bacterial inhibition was carried out using *Pseudomonas aeruginosa*, *Escherichia coli*, *Listeria monocytogenes*, *Salmonella typhimurium*, and *Campylobacter* strains. Table 1 reports the used experimental conditions and results. The decrease in microorganisms relative to initial concentrations and the control surface was determined. It is evident, with all considered strains, a significant inhibition of bacteria evolution from the considered active packaging. Microorganisms are used, those provided by the official method ISO 22196: 2011. The effectiveness is measured by comparing the survival of the bacteria in contact with the treated and untreated materials. Table 3 reports the experimental results.

The colony-forming units were determined with a microbiological counting technique and the antibacterial activity R = (Ut − U_0_) − (At − U_0_) was evaluated from the microbial count, which represents the elimination capacity expressed on a logarithmic basis along a period of 24 h of the bacteria in contact with the active material. The higher the R, the more the treated material has the ability to kill bacteria. U_0_, U_t_, and A_t_ were expressed as Log (CFU/cm^2^). The capability of bacteria inhibition from the salicylate present in the Nylon/PE-Active Filler pouches towards the analyzed strains ranged from 1 to 5 orders of magnitude higher than the untreated film, highlighting a significant activity with respect to *Pseudomonas* and *Campylobacter*; towards the other strains, despite slightly lower, there is still evidence of a bacteriostatic activity from the prepared active packaging.

**Figure 3:**
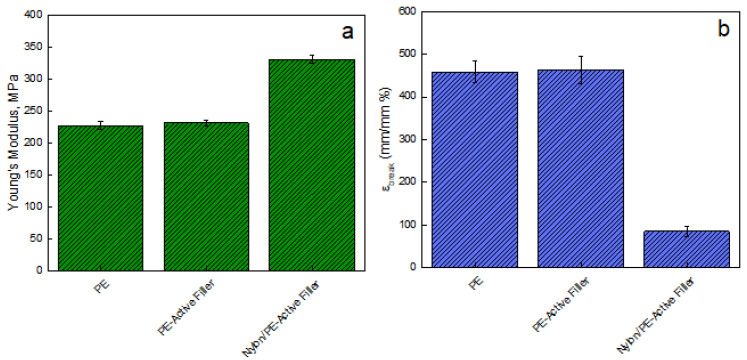
(**a**) Young’s Modulus (MPa) and (**b**) Elongation at break point (mm/mm%) evaluated on films of: pure PE, PE-Active Filler, and Nylon/PE-Active Filler.

Figure 3 reports the Young modulus [MPa] evaluated on films of unfilled PE, PE-Active Filler, and Nylon/PE-Active Filler. The introduction of the filler slightly improves the modulus of pure PE that passes from 228 ± 5.84 MPa to 233 ± 4.80 MPa; the modulus of the coupled active packaging (Nylon/PE-Active Filler) appears significantly improved, being 332 ± 6.25 MPa. This is an indication of good adhesion between the PE active layer and the Nylon external layer, fundamental for the pouches’ resistance. Besides, there is no noticeable difference concerning the elongation at break point for PE and PE-Active Filler since both show an ε_break_ equal to 460%. However, the coupling with Nylon film led to a substantial reduction of ε_break_ of about 81%.

**Figure 4:**
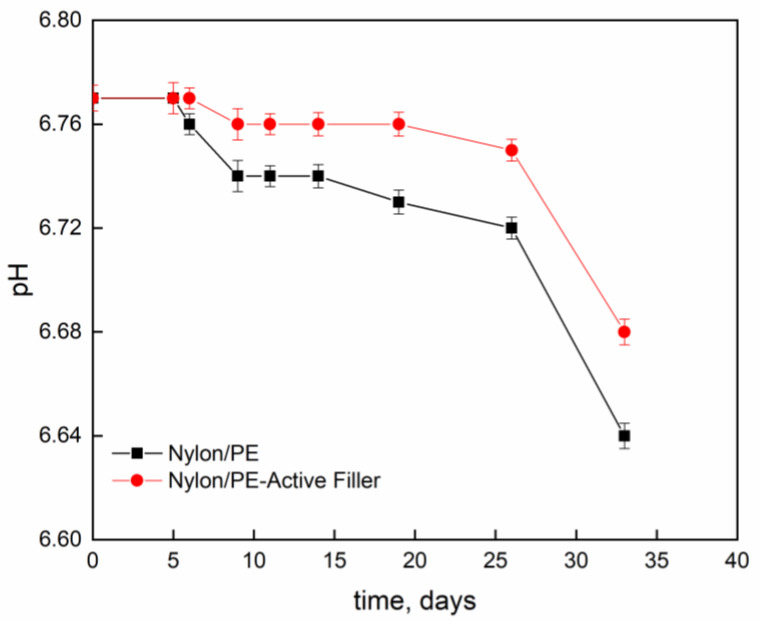
Evolution of pH, as function of time, of milk stored at 4 °C into Nylon/PE and Nylon/PE-Active Filler pouches.

**Figure 5:**
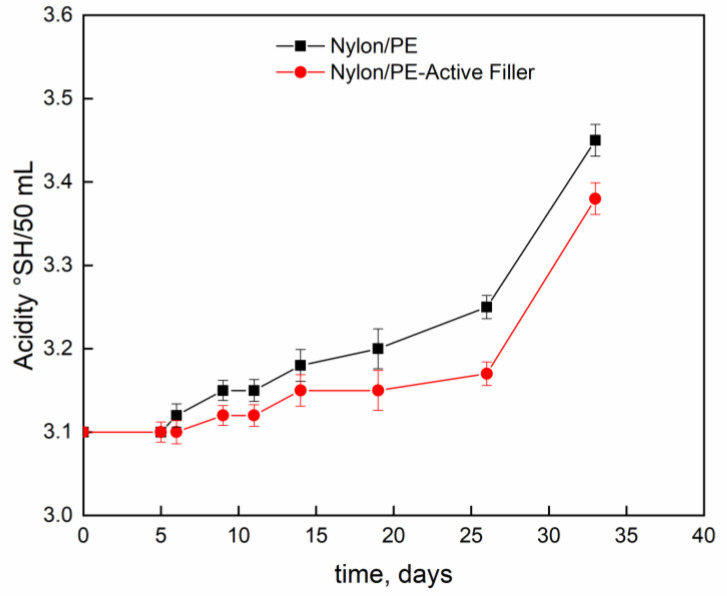
Evolution of acidity index (°SH/mL) as function of time, of milk stored at 4 °C into Nylon/PE and Nylon/PE-Active Filler pouches.

Figure 4 and Figure 5 report pH evolution and acidity index (°SH/mL) along the contact time evaluated on milk stored at 4 °C into Nylon/PE and Nylon/PE-Active Filler pouches. The monitoring of acidity level, during milk storage is essential for the control of dairy gel textural as well as organoleptic properties. As expected, the starting fresh milk’s pH shows a value slightly acidic. The pH decreases as function of time and, consequently the acidity index increases. It is well known that during milk fermentation lactic bacteria convert part of α- and β-lactoses into D- and L- lactic acids, leading to a pH decrease and, as a consequence, determining the coagulation of caseins [19]. Milk stored in the active packaging displays a drop in pH less pronounced than the one evidenced in the milk stored in pouches with no active filler. The presence of salicylate, that can migrate into the packed milk is able to neutralize the hydrogen ions in solution and allows the maintenance of pH at acceptable levels for a longer time.

The results concerning the total bacterial count evolution on packaged milk were reported as the log(CFU/mL) respect to the storage time useful for the application of Gompertz’s equation. 

The evaluated parameters from Equation (3), which are statistically significant, are reported in Table 4.

**Figure 6:**
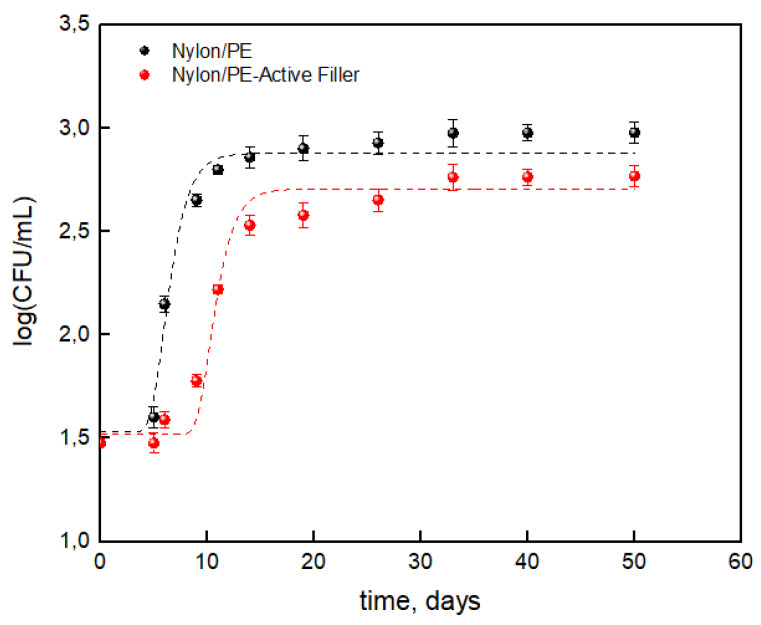
Log(CFU/mL) of TCB on packaged milk during storage time.

Figure 6 shows that the salicylate anion noticeably reduced the TBC during the storage time leading to a reduction of about 38% compared to the packaging without the active filler. Data reported in Table 4 show a noticeable difference between the two packaging, suggesting that the salicylate anion has an influence on maximum cell growth (μ_max_) and cell growth at stationary phase (A). The presence of the active filler led to a reduction of about 12% for cell growth at stationary phase and about 10% for maximum cell growth. Then it is worth observing a noticeable increase in lag time which roughly doubled for the Nylon/PE-Active Filler (from about 5 days to 9 days). The advantages conferred by the active filler can then state that it slowed the growth of total bacterial count during the storage allowing to extend the shelf life of milk from 6 days up to 10 days, respect to the Nylon/PE packaging without active filler.

## 4. Conclusions

The study reports on the fabrication of active pouches based on a coupled system Nylon/PE. The PE internal layer was loaded with LDH hosting salicylate ion, as antimicrobial (EC Directive 10/2011/EC of 14 January 2011). The intercalation of the active molecule into the LDH galleries was evaluated using either XRD analysis and by controlled release studies. The release kinetics showed a slow release of the salicylate bounded to the nano-carrier, compared with the same packaging in which the active molecule was free dispersed into the PE layer. The bacterial inhibition of the active layer against *Pseudomonas aeruginosa*, *Escherichia coli*, *Listeria monocytogenes*, *Salmonella typhimurium*, and *Campylobacter* was analyzed. Overall migration tests on the PE active layer demonstrated that the prepared active material is suitable for food contact, being the migration amount within the EU regulation limits. Fresh milk was packaged either into the active pouches, or in pouches based on pure polymers. pH evolution, acidity index (SH °/mL), and total bacteria count (TBC) were evaluated as function of the time at 4 °C. The decreasing of the pH resulted much slower for the milk packaged into the active pouches, due to the presence of the salicylate able to neutralize hydrogen ions produced from lactic acid. TBC evolution resulted greatly inhibited from the active packaging and shelf life (S.L.) was predicted through the Gompertz’s equation. Results evidenced a prolongation of the S.L. of milk packaged in active pouches from 6 to 10 days. 

## Figures and Tables

**Table 1:** Peppas–Sahlin’s model parameters obtained from Equation (1).

	PE-Active Filler	PE_Sal Free
K_1_ (days^−m^)	51.39 ± 2.36 ^a^	111 ± 1.96 ^b^
K_2_ (days^−2m^)	−6.51 ± 0.60 ^a^	−30.6 ± 1.26 ^b^
m	0.52 ± 0.03 ^a^	0.21 ± 0.01 ^b^
R^2^	0.995	0.992

^a^ and ^b^ are compact letters display; means having different superscript lowercase letters for a parameter are significantly different (*p* < 0.05) according to the Tukey test.

**Table 2:** Global migration from the active PE-Active Filler.

Simulant	D1 Ethanol at 50% (*v*/*v*)	Limits
	Global migration into aqueous food simulant by filling a container UNI EN 1186-1: 2003 + UNI EN 1186-9: 2003	
Temperature of the test	40 °C	
Contact time	10 days	
Global migration average in the simulant	8.3 (mg/dm^2^)	10 (mg/dm^2^)

**Table 3:** Inhibition of Pseudomonas, Escherichia coli, Listeria, Salmonella, and Campylobacter from the Nylon/PE-Active Filler pouches according to the directive ISO 22196: 2011.

Bacterial Strain	Pseudomonas Aeruginosa ATCC 15442	Escherichia Coli ATCC 8739	Listeria Monocytogenes ATCC 13932	Campylobacter ATCC 33559	Salmonella Enterica Typhimurium ATCC 14028
Sample size	50 × 50 (mm × mm)	40 × 40 (mm × mm)	50 × 50 (mm × mm)	50 × 50 (mm × mm)	50 × 50 (mm × mm)
Sample thickness	1.0 mm	0.070 mm	2.0 mm	1.0 mm	1.0 mm
Inoculum volume	0.4 mL	0.4 mL	0.4 mL	0.4 mL	0.4 mL
Number of bacteria available in the inoculum	410.000	25.000	380.000	300.000	120.000
Ut: Count of bacteria recovered from non-treated samples after 24 h from inoculation	5.3 (Log)	5.7 (Log)	4.8 (Log)	3.8 (Log)	4.3 (Log)
At: Count of bacteria recovered from treated samples after 24 h from inoculation	*n.d.	4.6 (Log)	3.0 (Log)	<0.4 (Log)	1.6 (Log)
Antibacterial activity R = (Ut − U_0_) − (At − U_0_) (ISO 22196: 2011)	>5.3	1.1	1.7	>3.8	2.6

* n.d. not detectable.

**Table 4:** Parameters derived from Equation (3).

	Nylon/PE-Active Filler	Nylon/PE
K	1.52 ± 0.07 ^a^	1.53 ± 0.04 ^a^
A	1.19 ± 0.18 ^a^	1.35 ± 0.20 ^b^
μ_max_ (days^−1^)	0.35 ± 0.02 ^a^	0.39 ± 0.01 ^b^
λ (days)	9 ± 1.16 ^a^	4.7 ± 2.05 ^b^
Shelf life (days)	10.2 ± 1.21 ^a^	6.05 ± 0.65 ^b^
R^2^	0.953	0.945

Means having different superscript lowercase letters for a parameter are significantly different (*p* < 0.05) through the Tukey test. ^a^ and ^b^ are compact letters display.

## Data Availability

Data available on request due to restrictions eg privacy or ethical.
